# Revisiting the concept of bout: associations of moderate-to-vigorous physical activity sessions and non-sessions with mortality

**DOI:** 10.1186/s12966-024-01631-5

**Published:** 2024-07-29

**Authors:** Tongyu Ma, John Sirard, Lin Yang, Ye Li, Sharon Tsang, Amy Fu

**Affiliations:** 1 Department of Rehabilitation Sciences, The Hong Kong Polytechnic University, Hong Kong, China; 2 Department of Kinesiology, University of Massachusetts, Amherst, MA USA; 3 School of Nursing, The Hong Kong Polytechnic University, Hong Kong, China; 4 The Hong Kong Polytechnic University, Hung Hom, Kowloon, Hong Kong SAR China

**Keywords:** Physical activity session, Bout, Pattern, Cardiovascular mortality, Cohort study, NHANES

## Abstract

**Introduction:**

Current physical activity guidelines recommend 150 min of moderate-to-vigorous physical activity (MVPA) for health benefits, regardless of the pattern of MVPA. However, MVPA that occurs in sessions (MVPA-S) may have different health implications compared to MVPA that is not accumulated in sessions (MVPA-nonS). This study aimed to investigate the associations of MVPA-S and MVPA-nonS with mortality.

**Methods:**

We conducted a cohort study of the National Health and Nutrition Examination Survey 2003–2006 (*n* = 5,658) with accelerometer-measured physical activity at baseline and mortality followed through December 31, 2019. A session was defined as a time window of 30 min or longer where the average intensity was at or above 2020 counts/minute. MVPA accumulated within such sessions was quantified as MVPA-S, while MVPA accumulated outside the sessions was quantified as MVPA-nonS. We examined the joint association of MVPA-S and MVPA-nonS by classifying the participants into four groups (both < 75 min/week [referent], MVPA-S ≥ 75 and MVPA-nonS < 75, MVPA-S < 75 and MVPA-nonS ≥ 75, and both ≥ 75). We used 75 min as the cut-point because it is half of the guideline-recommended MVPA volume where a strong MVPA-mortality association has been observed in previous studies, and because it was close to the median of MVPA-nonS (75 min/week was the 54th percentile), allowing a sufficient sample size in each group for testing statistical significance. The hazard ratios and 95% confidence intervals were estimated with adjustment for important confounders.

**Results:**

During 13.9 years of follow-up (74,988 person-years), there were 1,424 deaths, out of which 472 were related to cardiovascular diseases (CVD). Compared to the referent combination (both < 75), the hazard ratios in the other three combinations were 0.48 (0.33–0.69), 0.85 (0.71–1.01), and 0.45 (0.30–0.67) for all-cause mortality; and were 0.34 (0.17–0.70), 0.96 (0.69–1.33), and 0.40 (0.17–0.90) for CVD mortality, respectively. Results were largely consistent in the spline-based models, age- and sex-stratified analyses, complete-case analysis, competing risk analysis, and the analysis excluding deaths within two years of follow-up.

**Conclusion:**

In conclusion, MVPA accumulated in sessions that lasted at least 30 min was associated with significant reductions in all-cause and CVD-specific mortality risks. The health implications of MVPA that were not accumulated in such sessions warrant further investigation.

**Supplementary Information:**

The online version contains supplementary material available at 10.1186/s12966-024-01631-5.

## Introduction

The numerous benefits of engaging in regular physical activity have been demonstrated in compelling evidence from epidemiological research and randomized clinical trials (RCTs) [1]. Promoting an active, healthy lifestyle has become crucial in mitigating the escalating burden of chronic diseases and associated premature mortality. While the health-enhancing effect of increased physical activity volume is well-established, there is limited knowledge concerning the ideal pattern for accumulating physical activity.

Both the current American and the World Health Organization guidelines recommend a minimum of 150 min of moderate-to-vigorous physical activity (MVPA) per week to obtain health benefits, regardless of the MVPA pattern [2, 3]. The 2018 edition of the Physical Activity Guidelines for Americans [2], in contrast to its 2008 predecessor, specifically states that “adults are no longer required to engage in physical activity in bouts of at least 10 minutes”. This recommendation is primarily supported by epidemiological studies, which suggested that MVPA was associated with various health benefits irrespective of the bout length [4]. On the other hand, the most compelling evidence supporting the cause-effect relationship between physical activity and disease prevention is built on RCTs of exercise training, which were typically supervised MVPA sessions that lasted between 30 and 90 min [5–7]. The RCTs that did not emphasize sessions of MVPA seldom report improvements in hard clinical endpoints [8, 9]. This discrepancy between epidemiological studies and RCTs concerning the bout concept has not been appropriately addressed.

Since the inaugural statement that physical activity of any duration is beneficial, there was a surge of pilot studies comparing brief, intermittent physical activity versus isoenergetic continuous physical activity [10]. These small-scale clinical trials are predominantly short-term studies, focusing on biomarkers that are responsive to acute stimulus, such as glucose and triglycerides. However, few studies have indicated a cause-effect link between the brief bouts of MVPA and chronic disease prevention. Thus far, there isn’t consistent evidence supporting the plausibility that brief, sporadic MVPA could induce equivalent cellular responses (e.g., mitochondria biogenesis) and physiological adaptations (e.g., improved cardiac contractile function) that are observed in exercise training [11, 12], potentially due to a lack of substantial and sustained upregulation of aerobic metabolism. Therefore, it remains inconclusive whether the acute changes in biomarkers triggered by brief bouts of MVPA would result in long-term benefits on clinical endpoints.

The lack of coherent sets of evidence in the relevant literature indicates a need to revisit the concept of MVPA bouts. Traditionally, a bout of MVPA is defined as consecutive ten minutes of physical activity at moderate intensity or higher with no more than two minutes of interruption [13]. This definition does not accommodate intermittent sports with brief episodes of strenuous exertion interspaced by low-intensity activities, such as soccer and basketball. The traditional algorithm might not be able to differentiate those sports sessions from brief walking episodes that occur sporadically in the workplace.

While emerging evidence indicates potential cardiometabolic health benefits of light-intensity physical activity (LIPA), most physical activity guidelines does not include specific recommendations for LIPA due to the lack of high-quality experimental studies [14]. Thus, our study focused on MVPA while treating LIPA as a confounding variable. This study developed a novel algorithm to capture MVPA accumulated in sessions (MVPA-S) and MVPA not accumulated in sessions (MVPA-nonS) from accelerometer data and investigated the associations of MVPA-S and MVPA-nonS with all-cause and cardiovascular disease (CVD)-specific mortality. We hypothesized that MVPA that occurs in sessions (30 min or longer) would confer greater health benefits compared to MVPA that occurs in a brief, sporadic manner.

## Method

**Fig. 1:**
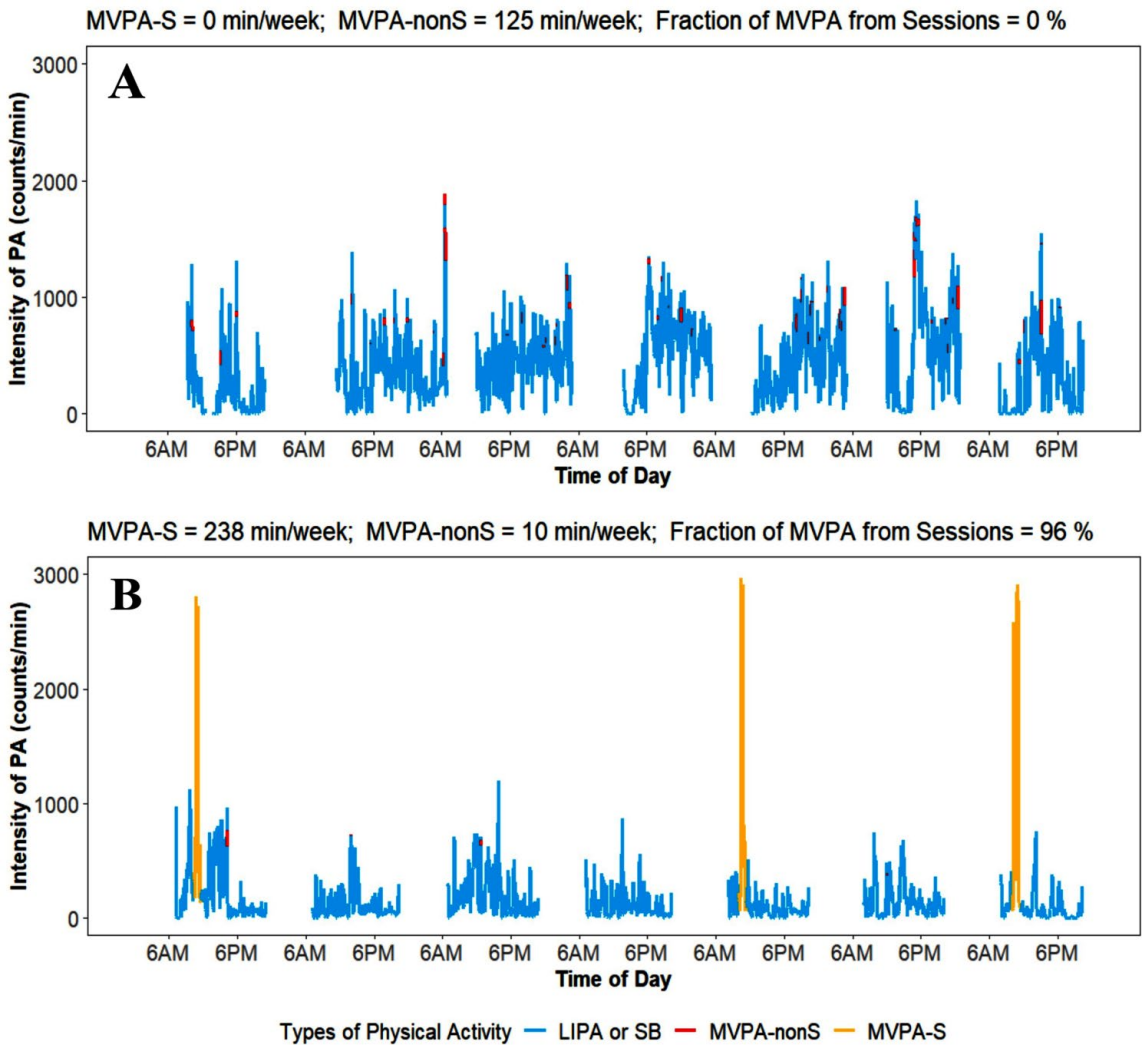
Illustration of MVPA-S and MVPA-nonS. **Panel A**: An individual with no MVPA accumulated in structured sessions. **Panel B**: An individual with MVPA predominantly accumulated in structured sessions. The intensity of physical activity (y-axis) represents the rolling mean of a ten-minute sliding window (average counts/minute over ten minutes). This was done to enhance the readability of the figure

The National Health and Nutrition Examination Survey (NHANES) is a cross-sectional survey of a nationally representative sample of the non-institutionalized population in the United States [15]. The vital status of NHANES participants was ascertained by a data linkage program between the survey participants and the death record in the National Death Index by matching their social security number, age, date of birth, etc. In this cohort study, we analyzed the association between baseline measures in the NHANES 2003–2006 cycles and mortality outcomes followed through December 31, 2019. After excluding participants aged < 20 years and those without valid data on vital status or accelerometer-measured physical activity, we had an eligible sample of 5,658 for the main analysis (sample diagram in Additional Fig. 1). We also conducted a complete-case analysis (*n* = 4,869) after excluding participants with missing covariates (See Fig. 1). 

### Accelerometer data processing

Participants were instructed to wear a uniaxial accelerometer (AM-7164, ActiGraph, FL, USA) for seven consecutive days except for sleep, bathing, and swimming. The intensity of human movement was saved as activity counts per minute (CPM) in the time-series file. We removed non-wear time defined as consecutive 60 min of zero CPM allowing for no more than two minutes above zero but below 100 CPM [13]. The remaining time was considered wear time. A valid day of accelerometer wear must have more than 10 h of wear time. To accurately reflect the habitual physical activity levels, we only included participants with four or more valid days of wear with at least one weekend day. We applied previously validated cut-off points for the categorization of intensity levels (< 100 CPM for sedentary behaviors [SB], 100–2019 CPM for LIPA, and ≥ 2020 CPM for MVPA) [16].

### Exposures

Landmark clinical trials on physical activity interventions predominantly involve supervised exercise sessions lasting at least 30 min. Examples include the 45- to 60-minute supervised sessions in the Diabetes Prevention Program [17], the 30- to 35-minute training sessions in the HF-ACTION trial [7], and the 60-minute group exercise in the trial comparing angioplasty with exercise interventions [6]. Furthermore, the American College of Sports Medicine’s Guidance for Prescribing Exercise recommends a daily session of 30–60 min of moderate-intensity aerobic training [18]. The high-quality evidence collectively suggests that an active, healthy lifestyle should include a minimum of a 30-minute session of activities per day at moderate intensity or higher.

To capture such sessions in free-living accelerometer data, we applied a sliding window algorithm to identify any periods of 30 min or longer where the average intensity within the window exceeded the moderate threshold (i.e., 2020 CPM). Also, the first and the last minute of a session must be at or above 2020 CPM to exclude the rest periods surrounding the MVPA sessions. The average intensity of the sliding window reflected the overall physical effort within the session. All MVPA accumulated within those captured windows was quantified as MVPA-S (MVPA accumulated in sessions). Conversely, MVPA accumulated outside of those windows was quantified as MVPA-nonS (MVPA not accumulated in sessions). During this process, one minute of vigorous-intensity physical activity (VPA) was equated to two minutes of moderate-intensity physical activity (MPA), in accordance with current guidelines [2, 3]. We developed an R algorithm using an adjustable sliding window to capture MVPA-S from the one minute-epoch, time-series data of accelerometry in the public NHANES database (see Additional Code).

Furthermore, we examined the validity of the proposed algorithm against ground-truth camera data. We found that the proposed algorithm outperformed the traditional algorithm in capturing MVPA sessions. The validation was conducted in a sample of 151 free-living individuals whose physical activity ground truth was verified using wearable camera data in the Capture-24 study [19]. Each individual worn the accelerometer for 24 h. We chose this dataset because it is, thus far, the largest of its kind that included concurrent accelerometer and ground-truth camera data. Although the Capture-24 study used wrist-worn accelerometers and the NHANES study placed accelerometers on the waist, our algorithm is not device-specific. It operates under the general assumption that higher acceleration indicates greater physical activity intensity, making the algorithm potentially applicable across different devices.

In the Capture-24 study, our algorithm captured 76 sessions of MVPA in leisure and transportation settings. We did not examine occupational MVPA or household MVPA because controversy exists regarding the health benefits of physical activities in those two settings [20–22]. The ground truth annotations for the captured sessions were in Additional Table 1. The median duration of ground truth MVPA within a single session is 38 min (interquartile range: 27–52 min), supporting that the algorithm captured what it purports to capture. Out of the 76 sessions, only 30 sessions were captured by the traditional algorithm, suggesting that the proposed algorithm was more effective than the traditional algorithm in capturing MVPA sessions (Table 1).

**Table 1:** Baseline characteristics across the joint classification of MVPA-S and MVPA-nonS. Data are mean (standard deviation) for continuous variables and number (percent) for categorical variables

	MVPA (minutes/week)				
	MVPA-S < 75 MVPA-nonS < 75 *n* = 2,973	≥ 75 < 75 *n* = 230	< 75 ≥ 75 *n* = 1,962	≥ 75 ≥ 75 *n* = 673	P-value
**Sociodemographic factors**					
Age (years)	56.9 (17)	53.7 (15.5)	42.9 (13.1)	42.1 (13.1)	< 0.01
Female (%)	1,706 (65.4)	148 (70.6)	805 (41.8)	209 (34.8)	< 0.01
Race/Ethnicity					
Non-Hispanic White (%)	1,606 (76.3)	156 (84.4)	961 (72.3)	351 (73.9)	< 0.01
Non-Hispanic Black (%)	540 (10.5)	23 (5.1)	375 (9.6)	116 (7.9)	
Mexican American (%)	490 (5.8)	36 (2.8)	483 (9.5)	148 (8.5)	
Other Hispanic (%)	47 (1.8)	6 (3.9)	64 (3.4)	26 (4.7)	
Other (%)	110 (5.6)	9 (3.9)	79 (5.2)	32 (5)	
Education					
Less than High School (%)	847 (19.6)	41(9.1)	476 (13.8)	149 (11.9)	< 0.01
High School Diploma (%)	745 (29.2)	44(19.9)	461 (24.5)	115 (15.7)	
College Education (%)	1,197 (51.1)	144 (70.8)	1,025 (61.7)	409 (72.4)	
Missing (%)	4 (0.1)	1 (0.2)	NA (NA)	NA (NA)	
**Behavioral variables**					
Healthy Eating Index	56.2 (14.2)	62.2 (13.9)	54.9 (13.6)	57.9 (13.9)	< 0.01
Tobacco Use (serum cotinine, ng/dL), %					
None (< 10)	2,126 (73.8)	203 (87.3)	1,430 (71.6)	528 (79.6)	< 0.01
Light (10–99)	107 (3.4)	7 (3.1)	114 (5.5)	44 (6.3)	
Moderate (100–299)	285 (12.4)	8 (4.3)	248 (14.1)	60 (8.4)	
Heavy (≥ 300)	171 (7.6)	3 (1.8)	118 (6.6)	30 (4.2)	
Missing (%)	104 (2.9)	9 (3.5)	52 (2.2)	11 (1.6)	
Alcohol Intake (grams/day), %					
Never (< 0.1)	2190 (77.3)	171 (73)	1367 (67.6)	421 (58.5)	< 0.01
Light (M < 28; F < 14)	207 (6.9)	16 (5.4)	176 (9.3)	76 (11.8)	
Moderate (M 28–56; F 14–28)	138 (5.7)	21 (9.3)	173 (9.6)	71 (14.2)	
Heavy (M ≥ 56; F ≥ 28)	145 (6.7)	16 (8)	171 (10.2)	74 (11.9)	
Missing (%)	113 (3.4)	6 (4.3)	75 (3.4)	31 (3.6)	

**Fig. 2:**
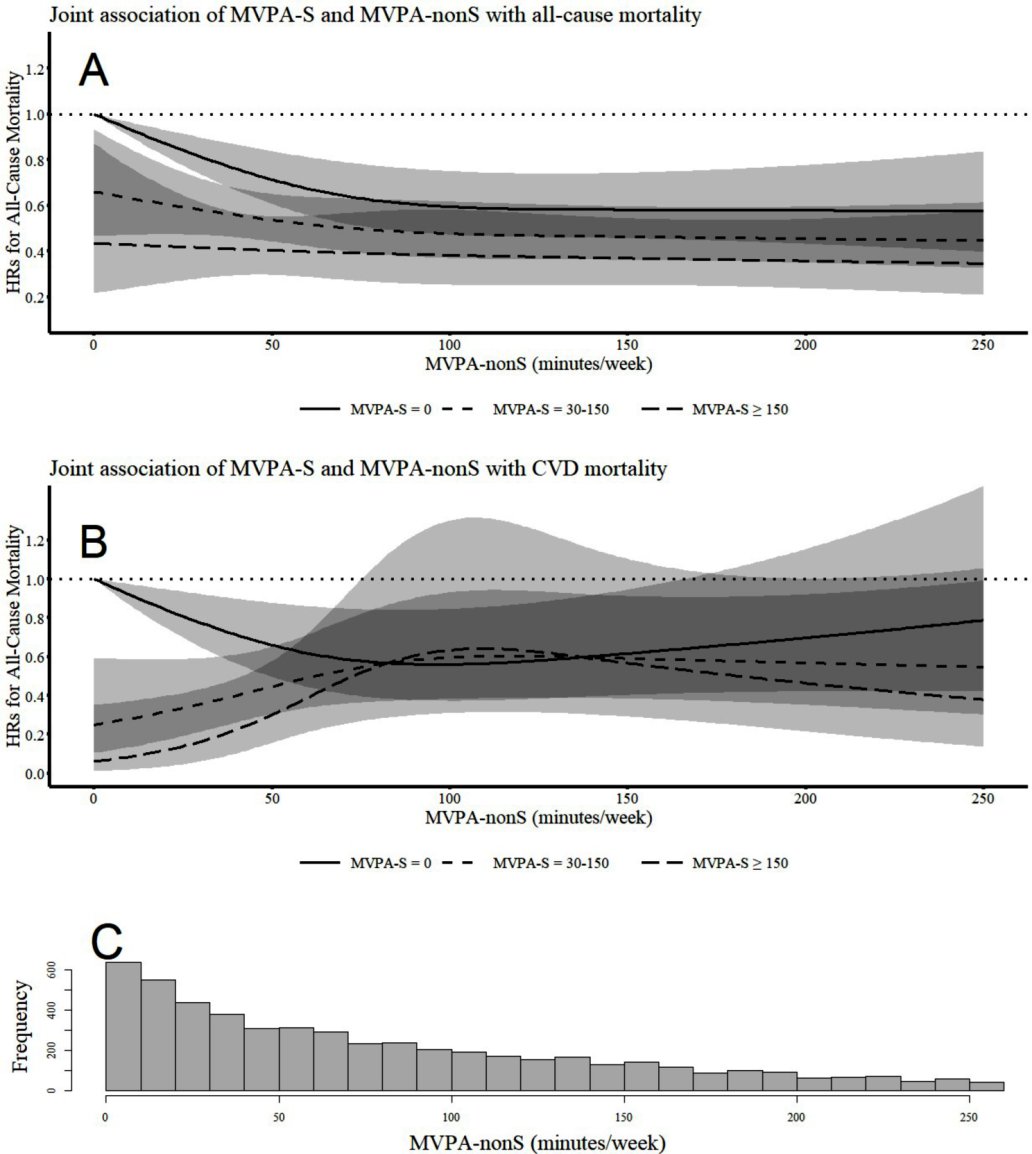
Joint association of MVPA-S and MVPA-nonS with mortality. The solid lines, dash lines and long dash lines are hazard ratios of mortality. The shaded areas are 95% confidence intervals. Adjusted for age, sex, ethnicity, education, diet quality, smoking, alcohol intake, light-intensity physical activity, body mass index, systolic blood pressure, emphysema, mobility disability, general health status, and diagnoses of diabetes, cardiovascular disease, and cancer. **Panel A**: All-cause mortality. **Panel B**: CVD mortality. **Panel C**: Histogram of MVPA-nonS

We presented three specific examples from the Capture-24 study to explain the reason why some sessions of MVPA were not recognized by the traditional algorithm (Additional Fig. 2). In the first example, an individual engaged in 90 min of hiking, which displayed a fluctuating pattern with many short LIPA/SB breaks. Because only 66% of the time was spent in MVPA, the traditional algorithm did not capture this hiking session. In the second example, an individual spent 80 min on a session of transportation walking, with several noticeable breaks lasting 2–5 min each. Because 71% of the time was spent in MVPA, the traditional algorithm did not capture this walking session. In the third example, an individual participated in 45 min of sports at a gym, following a high-intensity interval training style. This sport session was not captured by the traditional algorithm due to the frequent breaks of intermittent sports (See Fig. 2).

### Mortality Outcomes

The National Center for Health Statistics has released the most current Public-use Linked Mortality Files for NHANES 2003–2006 [23]. All-cause mortality was defined as death due to any reason. CVD-specific mortality was defined as death due to heart diseases (I00-I09, I11, I13, I20-I51) or cerebrovascular diseases (I60-I69) according to the International Classification of Diseases 10th version (ICD-10) in the NHANES mortality database. The duration of follow-up was calculated from the date of baseline examination measure to the date of death or December 31, 2019, whichever came first.

### Covariates

We selected covariates based on a previous investigation of NHANES 2003–2006 [24]. Demographic and behavioral confounders were assessed by questionnaires, including age in years at recruitment, sex (male/female), ethnicity (Non-Hispanic White, Non-Hispanic Black, Hispanic, and other), education level (less than high school, high school, and college or higher), smoking status (serum cotinine: <10, 10–100, 100–300, ≥ 300 ng/dL), alcohol consumption (males: <0.1, 0.1–28, 28–56, and ≥ 56; females: <0.1, 0.1–14, 14–28, and ≥ 28 g/day), healthy eating index (0-100 scale) [25], and LIPA (quartiles). Body mass index (BMI) (< 18.5, 18.6–24.9, 25-29.9, and ≥ 30 Kg/m^2^) and blood pressure were measured in the mobile examination centers. Self-reported medical history, such as physician-diagnosed chronic disease, was assessed during the in-person interview at home, including CVD (yes/no), diabetes (yes if diagnosed by physician, hemoglobin A1c ≥ 48 mmol/mol, or anti-diabetic medication usage), cancer (yes/no), emphysema (yes/no), mobility disability (yes/no), and self-rated general health (excellent, very good, good, fair, and poor).

### Statistical analysis

We fitted Cox proportional hazards models to investigate the joint association of MVPA-S and MVPA-nonS with mortality outcomes adjusting for confounders. Model zero was unadjusted. Model one adjusted for age, sex, ethnicity, education, diet quality, smoking, alcohol intake, and LIPA. Model two adjusted for model one plus BMI, systolic blood pressure, emphysema, mobility disability, general health status, and diagnoses of diabetes, CVD, and cancer.

We jointly classified the participants into four groups according to MVPA-S and MVPA-nonS levels (both < 75 min/week [referent], MVPA-S ≥ 75 and MVPA-nonS < 75, MVPA-S < 75 and MVPA-nonS ≥ 75, and both ≥ 75). We used 75 min as the cut-point because it is half of the guidelines-recommended MVPA volume where a strong MVPA-mortality association has been observed in previous studies [26], and because it was close to the median of MVPA-nonS (75 min/week was the 54th percentile), allowing a sufficient sample size in each group for testing statistical significance. Age- and sex-stratified analyses were conducted to examine whether the associations were modified by age (< 65 and ≥ 65 years) or sex.

In secondary analyses, we treated MVPA-nonS as a spline-based continuous variable with three knots at 25th, 50th, and 75^th^ percentiles to explore a potential dose-response relationship. We did not treat the MVPA-S as a continuous variable using restricted cubic spline because 65% of participants accumulated zero minutes of MVPA-S, making it challenging to apply the spline function. Thus, we categorized MVPA-S into three groups to explore a dose-response trend (0, 30–150, and ≥ 150 min/week).

In sensitivity analyses, we repeated the main analysis in the complete-case sample (*n* = 4,869). We also excluded deaths within two years of follow-up to account for the reverse causality bias. Additionally, we conducted competing risk analysis for CVD mortality based on the Fine & Gray model to account for the bias of competing events [27]. Given the growing evidence on the detrimental effects of SB on human health, we further adjusted for time spent on SB. Lastly, we further adjusted for total MVPA (MVPA-S plus MVPA-nonS) in the main analysis and applied a bout definition of at least 30 min with up to 6-minute interruptions (a scaled-up version of the standard 10-minute bout definition). The proportional hazards assumption was tested by visual inspections of the Schoenfeld residuals. Missing covariates were imputed using multiple imputations by chained equations (five imputations) [28]. Results were pooled according to the Rubin’s rule [29]. Statistical significance was determined at a two-sided alpha value of 0.05 for all tests. Analyses were conducted using R Studio survey package in June 2024.

## Results

MVPA-S presented a right-skewed distribution with a median of 0 min/week (interquartile range: 0 to 34 min/week). MVPA-nonS also followed a right-skewed distribution with a median of 68 min/week (interquartile range: 25 to 139 min/week) (Additional Figs. 3 and 4). There was a weak correlation between MVPA-S and MVPA-nonS (*r* = 0.25, *P* < 0.001) (Additional Fig. 5). In participants whose MVPA-S was above zero, the median number of MVPA sessions over the seven days of accelerometer wear was two. The median length of an entire session was 34 min. The overall composition of captured sessions was 69.1% MVPA, 24.2% LIPA, and 6.7% SB.

The baseline characteristics across the joint classifications of MVPA-S and MVPA-nonS are presented in Table 1. Age was inversely associated with both MVPA-S and MVPA-nonS. Participants in the group with lower MVPA-S and lower MVPA-nonS were older than those in other groups. There were more females than males among those with higher MVPA-S and lower MVPA-nonS. On the other hand, there were less females than males among those with higher MVPA-nonS and lower MVPA-S.

Table 1 is larger than one A4 or Letter page in length and it is placed at the end of the document.

During 13.9 years of follow-up (74,988 person-years), there were 1,424 deaths, out of which 472 were CVD-specific. In model one (Table 2), compared to the common reference (MVPA-S < 75 and MVPA-nonS < 75 min/week), accumulating 75 min/week of MVPA-S was associated with greater reductions in all-cause and CVD mortality compared to accumulating 75 min/week of MVPA-nonS. In model two (Table 2), further adjustment for biomarkers and chronic conditions attenuated the observed associations, especially for the group with lower MVPA-S and higher MVPA-nonS. However, the group with higher MVPA-S and lower MVPA-nonS was less affected.

**Table 2:** Joint associations of MVPA-S and MVPA-nonS with mortality. Data for mortality are hazard ratios and 95% confidence intervals. Model 0 was unadjusted. Model 1 adjusted for age, sex, ethnicity, education, diet quality, smoking, alcohol intake, and light-intensity physical activity. Model 2 adjusted for model 1 plus body mass index, systolic blood pressure, emphysema, mobility disability, general health status, and diagnoses of diabetes, cardiovascular disease, and cancer. * total MVPA equals the sum of MVPA-S and MVPA-nonS

MVPA (minutes/week)			All-cause mortality			CVD mortality		
MVPA session	MVPA non-session	MVPA* total	Model 0	Model 1	Model 2	Model 0	Model 1	Model 2
Category (Median)	Category (Median)	(Median)	No. of events / n 1,424 / 5,658			No. of events / n 472 / 5,658		
< 75 (0)	< 75 (30)	(32)	1 (ref) 1,143/2,793	1 (ref)	1 (ref)	1 (ref) 384/2,793	1 (ref)	1 (ref)
≥ 75 (148)	< 75 (49)	(195)	0.30 0.22–0.41 46/230	0.47 0.34–0.64	0.48 0.33–0.69	0.20 0.10–0.39 13/230	0.33 0.17–0.67	0.34 0.17–0.70
< 75 (0)	≥ 75 (140)	(157)	0.20 0.17–0.23 201/1,962	0.76 0.62–0.93	0.85 0.71–1.01	0.18 0.13–0.24 64/1,962	0.83 0.58–1.18	0.96 0.69–1.33
≥ 75 (151)	≥ 75 (175)	(361)	0.09 0.06–0.13 34/673	0.38 0.26–0.56	0.45 0.30–0.67	0.06 0.03–0.13 11/673	0.31 0.14–0.68	0.40 0.17–0.90

In the dose-response analyses, higher levels of MVPA-S were consistently associated with a lower risk of all-cause mortality regardless of the level of MVPA-nonS. On the other hand, the beneficial association between MVPA-nonS and all-cause mortality was mainly evident among those with zero MVPA-S, but not among those who had at least one session of MVPA-S. Similarly, the beneficial association between MVPA-nonS and CVD mortality was also only observed among those with zero MVPA-S. The lowest hazard of CVD mortality was observed in those achieving ≥ 150 min/week of MVPA-S combined with minimal levels of MVPA-nonS.

In age- and sex-stratified analyses, accumulating 75 min/week of MVPA-S was consistently associated with greater reductions in mortality risks compared to accumulating 75 min/week of MVPA-nonS (Additional Tables 2 and 3). In sensitivity analyses, outcomes remained largely unchanged when performing complete-case analysis (Additional Table 4), excluding deaths within two years of follow-up (Additional Table 5), and conducting competing risk analyses (Additional Table 6). Further adjusting for time spent on SB did not change the outcomes (Additional Table 7). Further adjusting for total MVPA substantially attenuated most associations, but the group with higher levels of MVPA-S and lower levels of MVPA-nonS was less affected (Additional Table 8). Applying the algorithm of 30 min with 6-minute interruptions enhanced the cardiovascular benefits of combining higher levels of MVPA-S and lower levels of MVPA-nonS (Additional Table 9).

## Discussion

In this population-based cohort study, we introduced an innovative method of categorizing total MVPA into MVPA accumulated in sessions and brief, sporadic MVPA that did not constitute sessions. These two forms of MVPA exhibited a weak correlation (*r* = 0.25, *P* < 0.001), indicating that they represent distinct types of human movement. Our findings highlight the beneficial association between MVPA accumulated in sessions and mortality risks. Meeting half of the guideline-recommended volume through MVPA-S (i.e., 75 min/week) was associated with a 52–55% reduction in all-cause mortality and a 60–66% reduction in CVD mortality, irrespective of the amount of MVPA-nonS. However, meeting half of the guideline-recommended volume through MVPA-nonS alone was not associated with significant reductions in mortality risks. In the secondary analyses, we found that the benefits of MVPA-nonS were mainly evident in those who had zero minutes of MVPA-S, while MVPA-nonS did not provide discernible benefits among those who already adhered to the current physical activity guidelines through MVPA-S (≥ 150 min/week). Interestingly, we found that achieving the current physical activity guidelines through MVPA-S while accumulating minimal levels of MVPA-nonS (e.g., less than 50 min/week) appeared to be the optimal pattern for cardiovascular health, which was associated with more than 80% reductions in CVD mortality compared to those accumulating zero minutes of any types of MVPA.

When applying the traditional algorithm of 30 min with 6-minute interruptions, the favorable CVD outcome of combining higher MVPA-S and lower MVPA-nonS was further enhanced, resulting in a nearly 90% risk reduction in CVD mortality in this group compared to the group with lower MVPA-S and lower MVPA-nonS. This evidence supports the selection of 30 min as the time window for capturing MVPA sessions. A major difference between the two algorithms is that the traditional algorithm requires 80% of all minutes stay above 2020 CPM, whereas the novel algorithm is more flexible and more capable of capturing a diverse range of MVPA sessions. As observed in the Capture-24 study, many MVPA sessions in free-living environments exhibited greater variability and complexity than the traditional 80% rule-of-thumb allows. It appears that, unlike lab-based treadmill walking or stationary cycling, free-living MVPA does not necessarily maintain a constant speed or physical effort during sessions of hiking, gym sports, or transportation walking.

Our findings agree with the landmark clinical trials in the prevention of diabetes and the secondary prevention of cardiovascular events. Those large-scale clinical trials on diabetes prevention have provided convincing and promising evidence of a cause-effect relationship between physical activity sessions and reductions in diabetes incidence [5, 30]. While the impact of physical activity sessions on reducing cardiovascular incidence among healthy individuals has not been tested in RCTs, a meta-analysis of RCTs involving patients with pre-existing coronary heart disease supported the effectiveness of physical activity sessions in reducing cardiovascular mortality [31].

To explore the mechanism of our findings, we tested the impact of MVPA-S and MVPA-nonS on cardiorespiratory fitness, as measured by sub-maximal exercise testing. Among participants with data on estimated maximal oxygen consumption (VO_2Max_) (*n* = 598), the partial correlation between MVPA-S and VO_2Max_ (*r* = 0.17, *P* < 0.01) was stronger than that between MVPA-nonS and VO_2Max_ (*r* = 0.05, *P* = 0.2) adjusting for age, sex, and BMI. We speculate that the strong associations between MVPA-S and mortality outcomes may be mediated by the improved fitness resulting from regular engagement in MVPA sessions. This speculation is supported by the comparison between our model one and model two: the benefits of higher MVPA-S and lower MVPA-nonS on mortality risks were less attenuated (only a 1% change in hazard ratios) by further adjustment for biomarkers, suggesting that MVPA-S had additional health benefits beyond the modification of risk factors such as systolic blood pressure, lipids, BMI, etc. Although performing brief, sporadic MVPA to interrupt sedentary time has been shown to reduce glucose, triglycerides, and blood pressure [10], whether it can increase VO_2Max_ remains to be researched. Sufficiently powered, multicenter, randomized clinical trials are needed to directly compare the effects of MVPA-S and MVPA-nonS on fitness and clinical endpoints.

We found that MVPA-S consisted of 95% MPA and 5% VPA, while MVPA-nonS contained 99% MPA and 1% VPA. However, we believe that the difference in VPA proportions is unlikely to be the main mechanism for the observed outcomes. First, the proportions of VPA were low in both types of MVPA. Furthermore, previous research has indicated that MPA and VPA may offer comparable benefits in reducing mortality risk when the total volume of activity is equivalent [32].

An example of higher MVPA-S combined with lower MVPA-nonS is an office worker with a sedentary job who regularly participates in leisure-time physical activity, such as several games of tennis per week. According to the mortality outcomes in our analysis, this lifestyle would be more beneficial for health than spending a high amount of time on brief, sporadic MVPA but engaging in limited MVPA sessions. Notably, the group with higher MVPA-S and lower MVPA-nonS spent more time on SB than the other groups (Additional Table 7). We speculate that engaging in MVPA-S may counteract the detrimental effects of SB [33]. Our findings provide a novel perspective on promoting physical activity: an active, healthy lifestyle for benefiting human health may not strictly depend on the total volume of MVPA. Instead, MVPA accumulated in sessions could be a better metric of health-enhancing physical activity than the traditional total MVPA.

We found that MVPA-nonS was beneficially linked to all-cause and CVD mortality only in the absence of MVPA-S. Our findings align well with a recent study of the UK Biobank cohort, which suggested that intermittent, brief episodes of MVPA lasting a few minutes were associated with a decreased risk of cardiovascular incidence and mortality among participants reporting no structured exercise [34]. An added insight from our analysis is that the maximal benefits of MVPA-nonS was observed at approximately 100 min/week. Performing additional MVPA-nonS beyond this point provided no further reductions in mortality risks.

Our findings do not contradict the previous evidence that bouts are not essential for eliciting health benefits [4]. Indeed, bouts and sessions are two separate concepts in this context. Bouts primarily concern the continuity of MVPA, while sessions focus on the overall physical effort of MVPA that is concentrated within a specific time frame to allow sufficient utilization of aerobic metabolism and activate the molecular pathways for favorable adaptations [11]. A session does not necessarily have to be continuous or bouted. For example, high-intensity interval training, which is non-bouted, can enhance cardiorespiratory fitness [35], supporting the rationale of our algorithm. The well-documented health benefits of intermittent sports also favor the plausibility of our findings [36].

Limitations of our study include the relatively small sample size compared to other cohorts and the lack of repeated measures of physical activity during the follow-up period. The estimates in our study were based on the assumption that the effect of physical activity does not change over time, known as the proportional hazards assumption. Although we did not identify statistical violations of this assumption, it is still possible that the effects of the two types of MVPA, along with their impact on health, may change during the 14-year follow-up period. Another limitation of our study is the paucity of information from accelerometry data regarding the purpose of MVPA and the environment in which MVPA was performed. Physical activity can be categorized into four domains based on its purpose, including recreational, occupational, transportation, and domestic physical activity. Previous studies suggest that recreational and transportation physical activities are health-enhancing while occupational and domestic activities are ineffectual or even detrimental [20–22]. Additionally, environmental factors such as air pollution have been shown to increase oxidative stress and inflammation which can counteract the benefits of physical activity [37]. Therefore, it warrants further investigation to determine whether the favorable health outcome of MVPA-S is driven by the physical activity pattern per se or driven by it being conducted as leisure-time activity, avoiding the potential psychological stress of work-related physical activity and/or negative environmental factors.

Another limitation of our study is the small sample size in the group with higher MVPA-S and lower MVPA-nonS (*n* = 230), resulting in wider confidence intervals in the estimation of mortality outcomes. This group was predominantly Non-Hispanic White females. The representativeness of the group and the generalizability of our findings need further investigations. Notably, this group had the highest education level, the highest diet quality, and the lowest smoking rates, depicting an overall healthier lifestyle than other groups. Whether the observed association is driven by a synergistic association of combining multiple healthy behaviors or just the pattern of MVPA also needs further clarifications. Causal inference cannot be drawn from epidemiological research. It is impossible to completely rule out all confounders in the causal path by conducting statistical adjustment. Additionally, reverse causality also cannot be ruled out, as it could well be that healthy individuals are more inclined to participate in MVPA sessions rather than MVPA sessions being the cause of their good health.

In conclusion, MVPA accumulated in sessions that lasted at least 30 min was associated with significant reductions in mortality risks, while the benefits of MVPA not accumulated in such sessions were mainly evident in those who had zero MVPA sessions. Whether the health implications of the two types of MVPA are substantially different warrants further investigation. If confirmed, future guidelines may consider providing separate recommendations for MVPA accumulated in sessions and MVPA not accumulated in sessions.

### Electronic supplementary material

Below is the link to the electronic supplementary material.

Supplementary Material 1

Supplementary Material 2

Supplementary Material 3

Supplementary Material 4

Supplementary Material 5

Supplementary Material 6

Supplementary Material 7

Supplementary Material 8

Supplementary Material 9

Supplementary Material 10

Supplementary Material 11

Supplementary Material 12

Supplementary Material 13

Supplementary Material 14

## Data Availability

The datasets analyzed during the current study are available in the NHANES repository, https://wwwn.cdc.gov/nchs/nhanes/.

## Abbreviations

BMI: Body mass index
CVD: Cardiovascular disease
CPM: Counts per minute
ICD-10: International Classification of Diseases 10th version
LIPA: Light-intensity physical activity
MPA: Moderate-intensity physical activity
MVPA: Moderate-to-vigorous physical activity
MVPA-nonS: MVPA not accumulated in sessions
MVPA-S: MVPA accumulated in sessions
NHANES: National Health and Nutrition Examination Survey
RCTs: Randomized clinical trials
SB: Sedentary behavior
VPA: Vigorous-intensity physical activity

## References

[1] Dishman RK, Heath GW, Schmidt MD, Lee IM. Physical activity epidemiology. 3rd ed. Human Kinetics; 2021.

[2] Piercy KL, Troiano RP, Ballard RM, Carlson SA, Fulton JE, Galuska DA, et al. The physical activity guidelines for americans. JAMA. 2018;320:2020–8. 30418471 10.1001/jama.2018.14854 PMC9582631

[3] Bull FC, Al-Ansari SS, Biddle S, Borodulin K, Buman MP, Cardon G, et al. World Health Organization 2020 guidelines on physical activity and sedentary behaviour. Br J Sports Med. 2020;54:1451–62. 33239350 10.1136/bjsports-2020-102955 PMC7719906

[4] Jakicic JM, Kraus WE, Powell KE, Campbell WW, Janz KF, Troiano RP, et al. Association between bout duration of physical activity and health: systematic review. Med Sci Sports Exerc. 2019;51:1213–9. 31095078 10.1249/MSS.0000000000001933 PMC6527142

[5] Diabetes Prevention Program Research Group. Reduction in the incidence of type 2 diabetes with lifestyle intervention or metformin. N Engl J Med. 2002;346:393–403. 11832527 10.1056/NEJMoa012512 PMC1370926

[6] Hambrecht R, Walther C, Möbius-Winkler S, Gielen S, Linke A, Conradi K, et al. Percutaneous coronary angioplasty compared with exercise training in patients with stable coronary artery disease: a randomized trial. Circulation. 2004;109:1371–8. 15007010 10.1161/01.CIR.0000121360.31954.1F

[7] O’Connor CM, Whellan DJ, Lee KL, Keteyian SJ, Cooper LS, Ellis SJ, et al. Efficacy and safety of exercise training in patients with chronic heart failure: HF-ACTION randomized controlled trial. JAMA. 2009;301:1439–50. 19351941 10.1001/jama.2009.454 PMC2916661

[8] Tang MS, Moore K, McGavigan A, Clark RA, Ganesan AN. Effectiveness of wearable trackers on physical activity in healthy adults: systematic review and meta-analysis of randomized controlled trials. JMIR mHealth uHealth. 2020;8:e15576. 32706685 10.2196/15576 PMC7407266

[9] Proper KI, Koning M, Van der Beek AJ, Hildebrandt VH, Bosscher RJ, van Mechelen W. The effectiveness of worksite physical activity programs on physical activity, physical fitness, and health. Clin J Sport Med. 2003;13:106–17. 12629429 10.1097/00042752-200303000-00008

[10] Loh R, Stamatakis E, Folkerts D, Allgrove JE, Moir HJ. Effects of interrupting prolonged sitting with physical activity breaks on blood glucose, insulin and triacylglycerol measures: a systematic review and meta-analysis. Sports Med. 2020;50:295–330. 31552570 10.1007/s40279-019-01183-w PMC6985064

[11] Drake JC, Wilson RJ, Yan Z. Molecular mechanisms for mitochondrial adaptation to exercise training in skeletal muscle. FASEB J. 2016;30:13. 26370848 10.1096/fj.15-276337 PMC6137621

[12] Seo DY, Kwak HB, Kim AH, Park SH, Heo JW, Kim HK, et al. Cardiac adaptation to exercise training in health and disease. Pflug Arch Eur J Phy. 2020;472:155–68. 10.1007/s00424-019-02266-3 31016384

[13] National Cancer Institute Web site. SAS programs for analyzing NHANES 2003–2004 accelerometer data. http://riskfactor.cancer.gov/tools/nhanes_pam. Accessed 25 May 2024.

[14] Chastin SF, De Craemer M, De Cocker K, Powell L, Van Cauwenberg J, Dall P, et al. How does light-intensity physical activity associate with adult cardiometabolic health and mortality? Systematic review with meta-analysis of experimental and observational studies. Br J Sports Med. 2019;53:370–6. 29695511 10.1136/bjsports-2017-097563 PMC6579499

[15] Centers for Disease Control and Prevention (CDC). National Center for Health Statistics (NCHS). National Health and Nutrition Examination Survey Data. Hyattsville, MD: U.S. Department of Health and Human Services, Centers for Disease Control and Prevention, https://www.cdc.gov/nchs/nhanes/index.htm. Accessed 25 May 2024.

[16] Troiano RP, Berrigan D, Dodd KW, Masse LC, Tilert T, McDowell M. Physical activity in the United States measured by accelerometer. Med Sci Sports Exerc. 2008;40:181. 18091006 10.1249/mss.0b013e31815a51b3

[17] Diabetes Prevention Program. Lifestyle Materials for Sessions 1–16. Standard Participant Handouts. https://dppos.bsc.gwu.edu/documents/1124073/1134992/LSMOP6.PDF/8098b30f-1acb-4546-8187-fd708985826f. Accessed 25 May 2024.

[18] Garber CE, Blissmer B, Deschenes MR, Franklin BA, Lamonte MJ, Lee IM, et al. Quantity and quality of exercise for developing and maintaining cardiorespiratory, musculoskeletal, and neuromotor fitness in apparently healthy adults: guidance for prescribing exercise. Med Sci Sports Exerc. 2011;43:1334–59. 21694556 10.1249/MSS.0b013e318213fefb

[19] Gershuny J, Harms T, Doherty A, Thomas E, Milton K, Kelly P, et al. Testing self-report time-use diaries against objective instruments in real time. Sociol Methodol. 2020;50:318–49. 10.1177/0081175019884591

[20] Fransson EI, Alfredsson LS, de Faire H, Knutsson U, Westerholm A. Leisure time, occupational and household physical activity, and risk factors for cardiovascular disease in working men and women: the WOLF study. Scand J Public Health. 2003;31:324–33. 14555368 10.1080/14034940210165055

[21] Holtermann A, Schnohr P, Nordestgaard BG, Marott JL. The physical activity paradox in cardiovascular disease and all-cause mortality: the contemporary Copenhagen General Population Study with 104 046 adults. Eur Heart J. 2021;42:1499–511. 33831954 10.1093/eurheartj/ehab087 PMC8046503

[22] Murphy MH, Donnelly P, Breslin G, Shibli S, Nevill AM. Does doing housework keep you healthy? The contribution of domestic physical activity to meeting current recommendations for health. BMC Public Health. 2013;13:1–6. 24139277 10.1186/1471-2458-13-966 PMC4016571

[23] National Center for Health Statistics Division of Analysis and Epidemiology. Continuous NHANES Public-use Linked Mortality Files, 2019. Hyattsville, Maryland. https://www.cdc.gov. Accessed 25 May 2024.

[24] Matthews CE, Troiano RP, Salerno EA, Berrigan D, Patel SB, Shiroma EJ, et al. Exploration of confounding due to poor health in an accelerometer-mortality study. Med Sci Sports Exerc. 2020;52:2546. 32472927 10.1249/MSS.0000000000002405 PMC7669589

[25] National Cancer Institute. Developing the Healthy Eating Index. https://epi.grants.cancer.gov/hei/developing.html. Accessed 25 May 2024.

[26] Ekelund U, Dalene KE, Tarp J, Lee IM. Physical activity and mortality: what is the dose response and how big is the effect? Br J Sports Med. 2020;54:1125–6. 31964630 10.1136/bjsports-2019-101765

[27] Fine JP, Gray RJ. A proportional hazards model for the sub-distribution of a competing risk. J Am Stat Assoc. 1999;94:496–509. 10.1080/01621459.1999.10474144

[28] Van Buuren S, Groothuis-Oudshoorn K. Mice: Multivariate imputation by chained equations in R. J Stat Softw. 2011;45:1–67. 10.18637/jss.v045.i03

[29] Rubin DB. Multiple imputation after 18 + years. J Am Stat Assoc. 1996;91:473–89. 10.1080/01621459.1996.10476908

[30] Li G, Zhang P, Wang J, Gregg EW, Yang W, Gong Q, et al. The long-term effect of lifestyle interventions to prevent diabetes in the China Da Qing diabetes Prevention Study: a 20-year follow-up study. Lancet. 2008;371:1783–9. 18502303 10.1016/S0140-6736(08)60766-7

[31] Anderson L, Thompson DR, Oldridge N, Zwisler AD, Rees K, Martin N, et al. Exercise-based cardiac rehabilitation for coronary heart disease. Cochrane Database Syst Rev. 2016;1:14651858. 10.1002/14651858.CD001800.pub3 PMC6491180 26730878

[32] Lopez JP, Sabag A, Juan MM, Rezende LF, Pastor-Valero M. Do vigorous-intensity and moderate-intensity physical activities reduce mortality to the same extent? A systematic review and meta-analysis. BMJ Open Sport Exerc Med. 2020;6:e000775. 10.1136/bmjsem-2020-000775 PMC7610342 33178440

[33] Ekelund U, Steene-Johannessen J, Brown WJ, Fagerland MW, Owen N, Powell KE, et al. Does physical activity attenuate, or even eliminate, the detrimental association of sitting time with mortality? A harmonised meta-analysis of data from more than 1 million men and women. Lancet. 2016;388:1302–10. 27475271 10.1016/S0140-6736(16)30370-1

[34] Ahmadi MN, Hamer M, Gill JM, Murphy M, Sanders JP, Doherty A, et al. Brief bouts of device-measured intermittent lifestyle physical activity and its association with major adverse cardiovascular events and mortality in people who do not exercise: a prospective cohort study. Lancet Public Health. 2023;8:e800–10. 37777289 10.1016/S2468-2667(23)00183-4

[35] Wen D, Utesch T, Wu J, Robertson S, Liu J, Hu G, et al. Effects of different protocols of high intensity interval training for VO2max improvements in adults: a meta-analysis of randomized controlled trials. J Sci Med Sport. 2019;22:941–7. 30733142 10.1016/j.jsams.2019.01.013

[36] Castillo-Bellot I, Mora-Gonzalez J, Fradua L, Ortega FB, Gracia-Marco L. Effects of recreational Soccer on Health outcomes: a narrative review. J Sci Sport Exerc. 2019;1:142–50. 10.1007/s42978-019-0012-9

[37] Giorgini P, Rubenfire M, Bard RL, Jackson EA, Ferri C, Brook RD. Air pollution and exercise. J Cardiopulm Rehabil Prev. 2016;36:84–95. 26378494 10.1097/HCR.0000000000000139

