# Supplementary figures and images for: Revisiting the concept of bout: associations of moderate-to-vigorous physical activity sessions and non-sessions with mortality
Source: Int J Behav Nutr Phys Act. 2024 Jul 29;21:81. doi: 10.1186/s12966-024-01631-5 (PMC11287937; doi:10.1186/s12966-024-01631-5)

**Additional Figure 1**. Sample diagram.


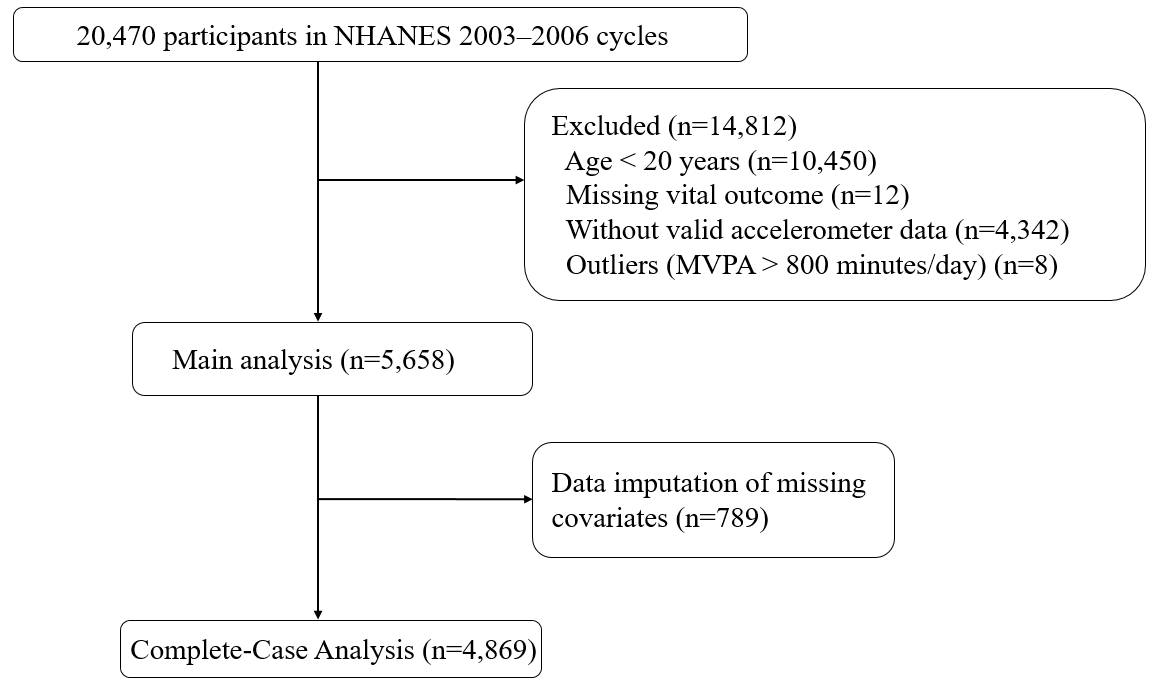

Supplement: Supplementary file 1 — Supplementary Material 1 [file 12966_2024_1631_MOESM1_ESM.docx]

**Additional Figure 3.** Histogram of MVPA-S.

**
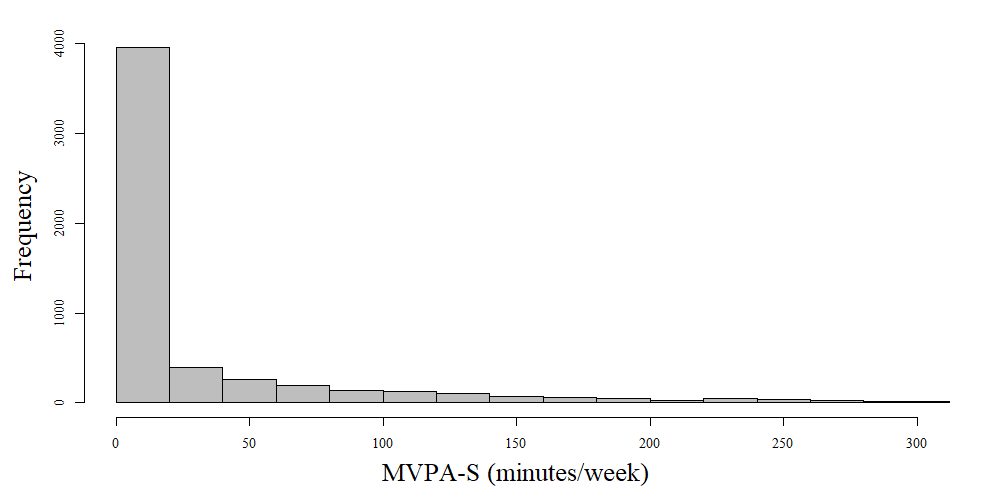
**

Supplement: Supplementary file 3 — Supplementary Material 3 [file 12966_2024_1631_MOESM3_ESM.docx]

**
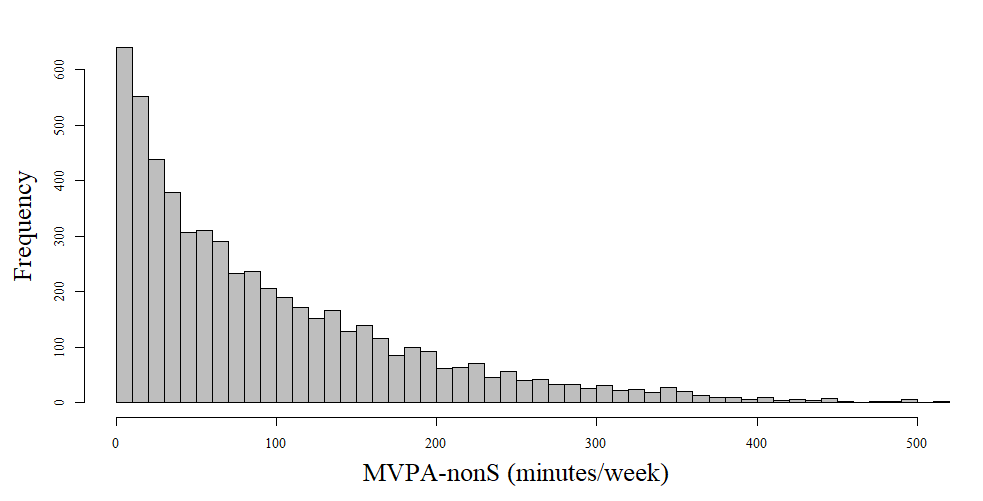
Additional Figure 4.** Histogram of MVPA-nonS.

Supplement: Supplementary file 4 — Supplementary Material 4 [file 12966_2024_1631_MOESM4_ESM.docx]

**
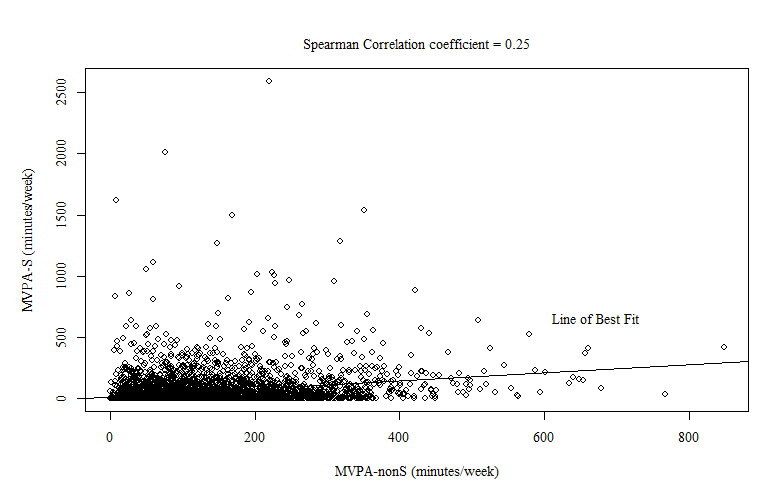
Additional Figure 5.** Correlation between MVPA-S and MVPA-nonS.

Supplement: Supplementary file 5 — Supplementary Material 5 [file 12966_2024_1631_MOESM5_ESM.docx]
