# Supplementary material for: Revisiting the concept of bout: associations of moderate-to-vigorous physical activity sessions and non-sessions with mortality
Source: Int J Behav Nutr Phys Act. 2024 Jul 29;21:81. doi: 10.1186/s12966-024-01631-5 (PMC11287937; doi:10.1186/s12966-024-01631-5)
Supplement: Supplementary file 2 — Supplementary Material 2 [file 12966_2024_1631_MOESM2_ESM.docx]

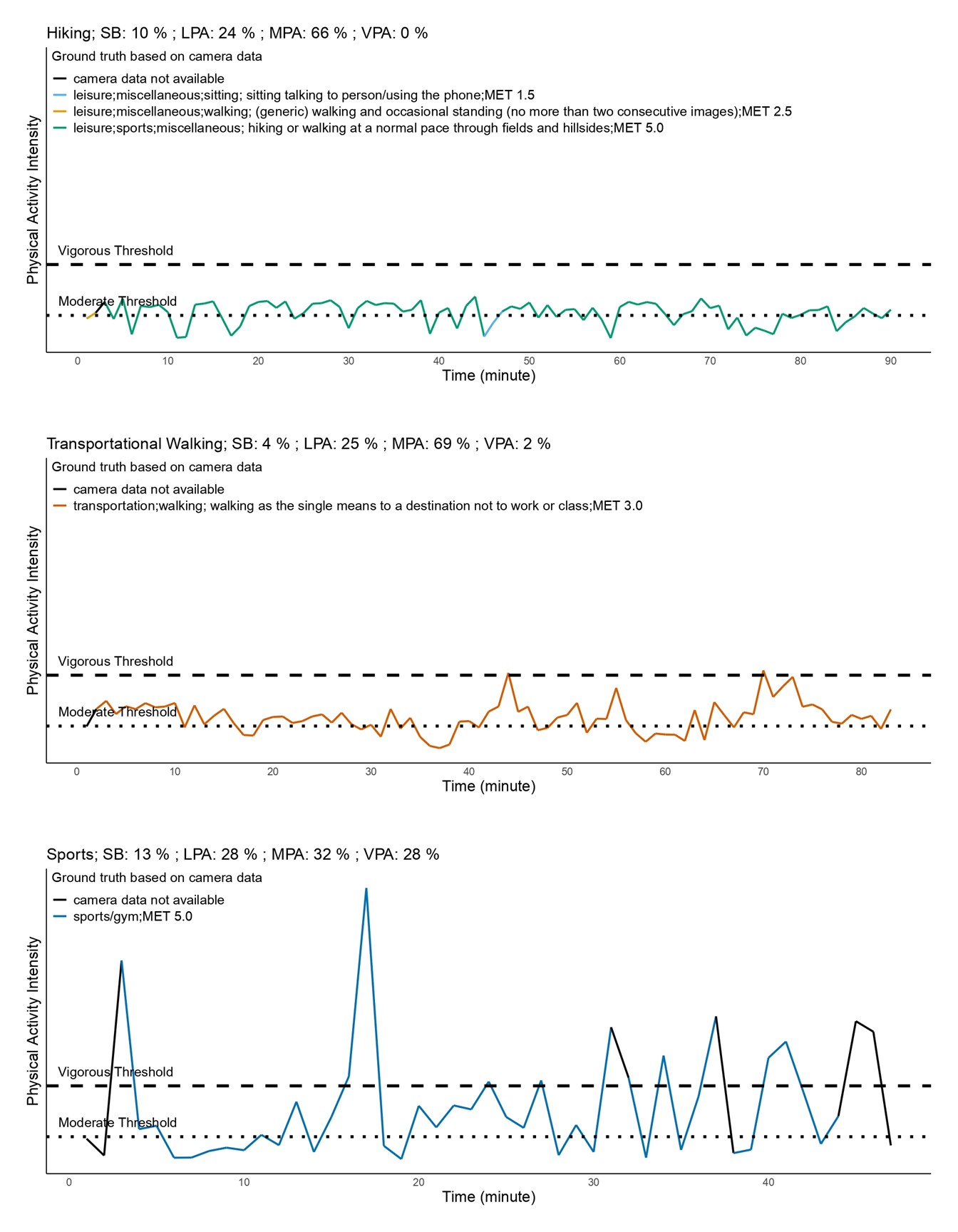
**Additional Figure 2.** Three sessions of MVPA that were captured by the proposed algorithm but not captured by the traditional algorithm.
