# Supplementary material for: Revisiting the concept of bout: associations of moderate-to-vigorous physical activity sessions and non-sessions with mortality
Source: Int J Behav Nutr Phys Act. 2024 Jul 29;21:81. doi: 10.1186/s12966-024-01631-5 (PMC11287937; doi:10.1186/s12966-024-01631-5)
Supplement: Supplementary file 6 — Supplementary Material 6 [file 12966_2024_1631_MOESM6_ESM.docx]

**Additional Table 1.** The camera data-based ground truth of MVPA in the captured sessions.

| **MVPA Sessions** | **The ground truth of MVPA accumulated in sessions** | **Whether captured by the traditional algorithm** |
| --- | --- | --- |
| 1 | transportation;private transportation; bicycling;MET 4.0 × 23 (minutes) | not captured |
|  | transportation;walking; walking as the single means to a destination not to work or class;MET 3.0 × 10 (minutes) |  |
| 2 | transportation;walking; walking as the single means to work or class (not from);MET 3.5 × 23 (minutes) | captured |
| 3 | transportation;private transportation; bicycling;MET 4.0 × 16 (minutes) | not captured |
|  | transportation;walking; walking as the single means to a destination not to work or class;MET 3.0 × 9 (minutes) |  |
| 4 | transportation;walking; walking as the single means to a destination not to work or class;MET 3.0 × 59 (minutes) | not captured |
| 5 | leisure;sports;gymnasium and athletics;athletics; running;MET 8.0 × 24 (minutes) | captured |
|  | sports/gym;MET 5.5 × 5 (minutes) |  |
|  | walking;MET 3.0 × 106 (minutes) |  |
| 6 | transportation;walking; walking as the single means to a destination not to work or class;MET 3.0 × 29 (minutes) | captured |
|  | transportation;walking; walking as the single means to work or class (not from);MET 3.5 × 19 (minutes) |  |
| 7 | sports/gym;MET 6.0 × 84 (minutes) | captured |
| 8 | leisure;miscellaneous;walking; loading /unloading a car implied walking;MET 3.5 × 1 (minutes) | captured |
|  | transportation;walking; walking as the single means to a destination not to work or class;MET 3.0 × 20 (minutes) |  |
| 9 | transportation;walking; walking as the single means to work or class (not from);MET 3.5 × 26 (minutes) | captured |
| 10 | transportation;private transportation; bicycling;MET 4.0 × 20 (minutes) | not captured |
| 11 | sports/gym;MET 5.5 × 5 (minutes) | captured |
|  | sports/gym;MET 6.0 × 9 (minutes) |  |
|  | transportation;private transportation; bicycling;MET 4.0 × 9 (minutes) |  |
| 12 | transportation;private transportation; bicycling;MET 4.0 × 31 (minutes) | not captured |
| 13 | transportation;private transportation; bicycling;MET 4.0 × 60 (minutes) | not captured |
| 14 | leisure;miscellaneous;walking; loading /unloading a car implied walking;MET 3.5 × 2 (minutes) | not captured |
|  | transportation;walking; walking as the single means to a destination not to work or class;MET 3.0 × 39 (minutes) |  |
| 15 | transportation;walking; walking as the single means to a destination not to work or class;MET 3.0 × 66 (minutes) | not captured |
| 16 | transportation;walking; walking as the single means to a destination not to work or class;MET 3.0 × 40 (minutes) | not captured |
| 17 | transportation;walking; walking as the single means to a destination not to work or class;MET 3.0 × 23 (minutes) | not captured |
| 18 | transportation;walking; walking as the single means to a destination not to work or class;MET 3.0 × 23 (minutes) | not captured |
| 19 | transportation;walking; walking as the single means to a destination not to work or class;MET 3.0 × 29 (minutes) | captured |
| 20 | sports/gym;MET 5.5 × 1 (minutes) | not captured |
|  | sports/gym;MET 8.5 × 3 (minutes) |  |
|  | sports/gym;MET 9.0 × 8 (minutes) |  |
|  | transportation;private transportation; bicycling;MET 4.0 × 15 (minutes) |  |
| 21 | transportation;private transportation; bicycling;MET 4.0 × 24 (minutes) | not captured |
| 22 | leisure;sports;miscellaneous; hiking or walking at a normal pace through fields and hillsides;MET 5.0 × 114 (minutes) | captured |
| 23 | leisure;sports;gymnasium and athletics;athletics; running;MET 8.0 × 23 (minutes) | captured |
|  | sports/gym;MET 5.5 × 9 (minutes) |  |
|  | transportation;private transportation; bicycling;MET 4.0 × 26 (minutes) |  |
| 24 | transportation;walking; walking as the single means to a destination not to work or class;MET 3.0 × 40 (minutes) | not captured |
| 25 | transportation;walking; walking as the single means to a destination not to work or class;MET 3.0 × 32 (minutes) | captured |
| 26 | transportation;walking; walking as the single means to a destination not to work or class;MET 3.0 × 54 (minutes) | not captured |
| 27 | transportation;walking; walking as the single means to a destination not to work or class;MET 3.0 × 31 (minutes) | not captured |
| 28 | transportation;private transportation; bicycling;MET 4.0 × 20 (minutes) | captured |
| 29 | sports/gym;MET 5.0 × 64 (minutes) | not captured |
|  | transportation;walking; walking as the single means to a destination not to work or class;MET 3.0 × 20 (minutes) | not captured |
| 30 | transportation;private transportation; bicycling;MET 4.0 × 40 (minutes) | not captured |
| 31 | transportation;private transportation; bicycling;MET 4.0 × 100 (minutes) | not captured |
| 32 | transportation;walking; walking as the single means to a destination not to work or class;MET 3.0 × 23 (minutes) | not captured |
|  | transportation;walking; walking as the single means to work or class (not from);MET 3.5 × 6 (minutes) | not captured |
| 33 | transportation;walking; walking as the single means to work or class (not from);MET 3.5 × 38 (minutes) | captured |
| 34 | transportation;private transportation; bicycling;MET 4.0 × 20 (minutes) | not captured |
| 35 | transportation;private transportation; bicycling;MET 4.0 × 23 (minutes) | not captured |
|  | transportation;walking; walking as the single means to a destination not to work or class;MET 3.0 × 8 (minutes) |  |
| 36 | transportation;walking; walking as the single means to a destination not to work or class;MET 3.0 × 88 (minutes) | captured |
| 37 | transportation;private transportation; bicycling;MET 4.0 × 37 (minutes) | captured |
| 38 | transportation;private transportation; bicycling;MET 4.0 × 41 (minutes) | captured |
| 39 | transportation;private transportation; bicycling;MET 4.0 × 41 (minutes) | not captured |
| 40 | transportation;private transportation; bicycling;MET 4.0 × 28 (minutes) | not captured |
| 41 | transportation;private transportation; bicycling;MET 4.0 × 52 (minutes) | not captured |
| 42 | leisure;sports;miscellaneous; hiking or walking at a normal pace through fields and hillsides;MET 5.0 × 48 (minutes) | captured |
| 43 | leisure;sports;miscellaneous; hiking or walking at a normal pace through fields and hillsides;MET 5.0 × 31 (minutes) | captured |
| 43 | sports/gym;MET 5.5 × 2 (minutes) | captured |
| 44 | leisure;sports;miscellaneous; hiking or walking at a normal pace through fields and hillsides;MET 5.0 × 87 (minutes) | not captured |
| 45 | walking;MET 3.0 × 29 (minutes) | captured |
| 46 | transportation;walking; walking as the single means to work or class (not from);MET 3.5 × 21 (minutes) | not captured |
| 47 | transportation;walking; walking as the single means to a destination not to work or class;MET 3.0 × 48 (minutes) | captured |
| 48 | leisure;sports;gymnasium and athletics;athletics; running;MET 8.0 × 25 (minutes) | captured |
| 49 | walking;MET 3.0 × 60 (minutes) | not captured |
| 50 | transportation;private transportation; bicycling;MET 4.0 × 20 (minutes) | not captured |
| 51 | transportation;private transportation; bicycling;MET 4.0 × 28 (minutes) | not captured |
| 52 | transportation;walking; walking as the single means to a destination not to work or class;MET 3.0 × 40 (minutes) | not captured |
| 53 | transportation;walking; walking as the single means to a destination not to work or class;MET 3.0 × 39 (minutes) | not captured |
| 54 | transportation;private transportation; bicycling;MET 4.0 × 24 (minutes) | captured |
|  | transportation;walking; walking as the single means to a destination not to work or class;MET 3.0 × 48 (minutes) |  |
| 55 | transportation;walking; walking as the single means to a destination not to work or class;MET 3.0 × 27 (minutes) | not captured |
| 56 | transportation;walking; walking as the single means to a destination not to work or class;MET 3.0 × 26 (minutes) | not captured |
| 57 | leisure;sports;gymnasium and athletics;athletics; running;MET 8.0 × 30 (minutes) | captured |
| 58 | walking;MET 3.0 × 80 (minutes) | captured |
| 59 | transportation;walking; walking as the single means to a destination not to work or class;MET 3.0 × 23 (minutes) | not captured |
| 59 | walking;MET 3.0 × 4 (minutes) | not captured |
| 60 | transportation;walking; walking as the single means to a destination not to work or class;MET 3.0 × 53 (minutes) | captured |
| 61 | sports/gym;MET 8.0 × 61 (minutes) | captured |
|  | transportation;walking; walking as the single means to work or class (not from);MET 3.5 × 8 (minutes) |  |
| 62 | transportation;walking; walking as the single means to a destination not to work or class;MET 3.0 × 7 (minutes) | not captured |
|  | walking;MET 3.0 × 30 (minutes) |  |
| 63 | transportation;walking; walking as the single means to a destination not to work or class;MET 3.0 × 6 (minutes) | not captured |
| 63 | walking;MET 3.0 × 16 (minutes) | not captured |
| 64 | walking;MET 3.0 × 38 (minutes) | not captured |
| 65 | transportation;walking; walking as the single means to a destination not to work or class;MET 3.0 × 39 (minutes) | not captured |
| 66 | leisure;miscellaneous;walking; walking upstairs;MET 4.0 × 2 (minutes) | not captured |
|  | transportation;walking; walking as the single means to a destination not to work or class;MET 3.0 × 31 (minutes) |  |
| 67 | leisure;sports;gymnasium and athletics;athletics; running;MET 8.0 × 22 (minutes) | captured |
|  | transportation;private transportation; bicycling;MET 4.0 × 5 (minutes) |  |
| 68 | transportation;walking; walking as the single means to a destination not to work or class;MET 3.0 × 84 (minutes) | captured |
| 69 | transportation;walking; walking as the single means to a destination not to work or class;MET 3.0 × 23 (minutes) | captured |
| 70 | transportation;walking; walking as the single means to a destination not to work or class;MET 3.0 × 29 (minutes) | not captured |
| 71 | transportation;walking; walking as the single means to a destination not to work or class;MET 3.0 × 22 (minutes) | not captured |
| 72 | transportation;private transportation; bicycling;MET 4.0 × 5 (minutes) | not captured |
|  | transportation;walking; walking as the single means to a destination not to work or class;MET 3.0 × 19 (minutes) |  |
| 73 | leisure;recreation;outdoor; walking/running playing with child(ren);MET 3.5 × 21 (minutes) | not captured |
| 73 | transportation;walking; walking as the single means to a destination not to work or class;MET 3.0 × 21 (minutes) | not captured |
| 74 | transportation;private transportation; bicycling;MET 4.0 × 23 (minutes) | not captured |
| 75 | sports/gym;MET 5.0 × 9 (minutes) | not captured |
|  | sports/gym;MET 6.0 × 4 (minutes) |  |
|  | transportation;private transportation; bicycling;MET 4.0 × 29 (minutes) |  |
| 75 | transportation;walking; walking as the single means to a destination not to work or class;MET 3.0 × 5 (minutes) |  |
| 76 | sports/gym;MET 3.5 × 49 (minutes) | not captured |
