# Supplementary material for: Revisiting the concept of bout: associations of moderate-to-vigorous physical activity sessions and non-sessions with mortality
Source: Int J Behav Nutr Phys Act. 2024 Jul 29;21:81. doi: 10.1186/s12966-024-01631-5 (PMC11287937; doi:10.1186/s12966-024-01631-5)
Supplement: Supplementary file 7 — Supplementary Material 7 [file 12966_2024_1631_MOESM7_ESM.docx]

**Additional Table 8.** Further adjusting for total MVPA (Total MVPA = MVPA-S + MVPA-nonS).

| **MVPA Session** | **MVPA non-Session** | **All-Cause Mortality** | **CVD Mortality** |
| --- | --- | --- | --- |
| <75 | <75 | 1 (ref) | 1 (ref) |
| ≥75 | <75 | 0.57  0.37-0.87 | 0.40  0.17-0.94 |
| <75 | ≥75 | 0.93  0.71-1.20 | 1.04  0.72-1.51 |
| ≥75 | ≥75 | 0.58  0.34-1.01 | 0.51  0.20-1.28 |
