# Supplementary material for: Revisiting the concept of bout: associations of moderate-to-vigorous physical activity sessions and non-sessions with mortality
Source: Int J Behav Nutr Phys Act. 2024 Jul 29;21:81. doi: 10.1186/s12966-024-01631-5 (PMC11287937; doi:10.1186/s12966-024-01631-5)
Supplement: Supplementary file 8 — Supplementary Material 8 [file 12966_2024_1631_MOESM8_ESM.docx]

**Additional Table 9.** Applying the algorithm of 30 minutes with 6-minute interruptions.

| **MVPA Session** | **MVPA non-Session** | **All-Cause Mortality** | **CVD Mortality** |
| --- | --- | --- | --- |
| <75 | <75 | 1 (ref) | 1 (ref) |
| ≥75 | <75 | 0.51  0.32-0.81 | 0.12  0.03-0.49 |
| <75 | ≥75 | 0.74  0.62-0.90 | 0.80  0.59-1.08 |
| ≥75 | ≥75 | 0.47  0.28-0.79 | 0.48  0.21-1.08 |
