# Supplementary material for: Revisiting the concept of bout: associations of moderate-to-vigorous physical activity sessions and non-sessions with mortality
Source: Int J Behav Nutr Phys Act. 2024 Jul 29;21:81. doi: 10.1186/s12966-024-01631-5 (PMC11287937; doi:10.1186/s12966-024-01631-5)
Supplement: Supplementary file 9 — Supplementary Material 9 [file 12966_2024_1631_MOESM9_ESM.docx]

**Additional Table 7.** Further adjusting for time spent on sedentary behavior.

| **MVPA Session** | **MVPA**  **non-Session** | **Time spent on sedentary behavior (hours/day)**  **Mean (standard deviation)** | **All-Cause Mortality** | **CVD Mortality** |
| --- | --- | --- | --- | --- |
| <75 | <75 | 8.20  (0.06) | 1 (ref) | 1 (ref) |
| ≥75 | <75 | 8.24  (0.10) | 0.48  0.33-0.69 | 0.34  0.16-0.70 |
| <75 | ≥75 | 7.19  (0.06) | 0.85  0.71-1.01 | 0.95  0.68-1.32 |
| ≥75 | ≥75 | 7.43  (0.10) | 0.45  0.30-0.67 | 0.39  0.17-0.89 |
