# Supplementary material for: Revisiting the concept of bout: associations of moderate-to-vigorous physical activity sessions and non-sessions with mortality
Source: Int J Behav Nutr Phys Act. 2024 Jul 29;21:81. doi: 10.1186/s12966-024-01631-5 (PMC11287937; doi:10.1186/s12966-024-01631-5)
Supplement: Supplementary file 10 — Supplementary Material 10 [file 12966_2024_1631_MOESM10_ESM.docx]

**Additional Table 2.** Age-stratified analysis.

|  |  | **All-Cause Mortality** | | **CVD Mortality** | |
| --- | --- | --- | --- | --- | --- |
| **MVPA**  **Session** | **MVPA**  **non-Session** | **<65 years** | **≥65 years** | **<65 years** | **≥65 years** |
| <75 | <75 | 1 (ref) | 1 (ref) | 1 (ref) | 1 (ref) |
| ≥75 | <75 | 0.47  0.24-0.90 | 0.52  0.35-0.78 | 0.26  0.03-2.01 | 0.38  0.18-0.80 |
| <75 | ≥75 | 0.89  0.70-1.15 | 0.80  0.58-1.10 | 1.06  0.55-2.06 | 0.86  0.53-1.40 |
| ≥75 | ≥75 | 0.49  0.25-0.96 | 0.45  0.25-0.82 | 0.42  0.11-1.58 | 0.49  0.17-1.41 |
