# Supplementary material for: Revisiting the concept of bout: associations of moderate-to-vigorous physical activity sessions and non-sessions with mortality
Source: Int J Behav Nutr Phys Act. 2024 Jul 29;21:81. doi: 10.1186/s12966-024-01631-5 (PMC11287937; doi:10.1186/s12966-024-01631-5)
Supplement: Supplementary file 11 — Supplementary Material 11 [file 12966_2024_1631_MOESM11_ESM.docx]

**Additional Table 3**. Sex-stratified analysis,

|  |  | **All-Cause Mortality** | | **CVD Mortality** | |
| --- | --- | --- | --- | --- | --- |
| **MVPA**  **Session** | **MVPA**  **non-Session** | **Male** | **Female** | **Male** | **Female** |
| <75 | <75 | 1 (ref) | 1 (ref) | 1 (ref) | 1 (ref) |
| ≥75 | <75 | 0.47  0.28-0.78 | 0.50  0.32-0.79 | 0.41  0.16-1.06 | 0.31  0.09-1.09 |
| <75 | ≥75 | 0.76  0.59-0.97 | 1.00  0.77-1.30 | 0.76  0.44-1.32 | 1.32  0.84-2.08 |
| ≥75 | ≥75 | 0.36  0.24-0.55 | 0.78  0.38-1.58 | 0.45  0.18-1.09 | 0.17  0.02-1.25 |
