# Supplementary material for: Revisiting the concept of bout: associations of moderate-to-vigorous physical activity sessions and non-sessions with mortality
Source: Int J Behav Nutr Phys Act. 2024 Jul 29;21:81. doi: 10.1186/s12966-024-01631-5 (PMC11287937; doi:10.1186/s12966-024-01631-5)
Supplement: Supplementary file 12 — Supplementary Material 12 [file 12966_2024_1631_MOESM12_ESM.docx]

**Additional Table 4.** Complete-case analysis (n=4,869).

| **MVPA Session** | **MVPA non-Session** | **All-Cause Mortality** | **CVD Mortality** |
| --- | --- | --- | --- |
| <75 | <75 | 1 (ref) | 1 (ref) |
| ≥75 | <75 | 0.48  0.33-0.70 | 0.35  0.17-0.71 |
| <75 | ≥75 | 0.85  0.71-1.01 | 0.96  0.69-1.34 |
| ≥75 | ≥75 | 0.45  0.30-0.67 | 0.40  0.17-0.91 |
