# Supplementary material for: Revisiting the concept of bout: associations of moderate-to-vigorous physical activity sessions and non-sessions with mortality
Source: Int J Behav Nutr Phys Act. 2024 Jul 29;21:81. doi: 10.1186/s12966-024-01631-5 (PMC11287937; doi:10.1186/s12966-024-01631-5)
Supplement: Supplementary file 13 — Supplementary Material 13 [file 12966_2024_1631_MOESM13_ESM.docx]

**Additional Table 5.** Excluding deaths within two years of follow-up (n=5,524).

| **MVPA Session** | **MVPA non-Session** | **All-Cause Mortality** | **CVD Mortality** |
| --- | --- | --- | --- |
| <75 | <75 | 1 (ref) | 1 (ref) |
| ≥75 | <75 | 0.47  0.32-0.68 | 0.37  0.18-0.76 |
| <75 | ≥75 | 0.87  0.72-1.06 | 1.02  0.74-1.40 |
| ≥75 | ≥75 | 0.43  0.28-0.64 | 0.33  0.13-0.85 |
