# Supplementary material for: Revisiting the concept of bout: associations of moderate-to-vigorous physical activity sessions and non-sessions with mortality
Source: Int J Behav Nutr Phys Act. 2024 Jul 29;21:81. doi: 10.1186/s12966-024-01631-5 (PMC11287937; doi:10.1186/s12966-024-01631-5)
Supplement: Supplementary file 14 — Supplementary Material 14 [file 12966_2024_1631_MOESM14_ESM.docx]

**Additional Table 6.** Competing risk analysis.

| **MVPA Session** | **MVPA non-Session** | **CVD Mortality** |
| --- | --- | --- |
| <75 | <75 | 1 (ref) |
| ≥75 | <75 | 0.63  0.30-1.34 |
| <75 | ≥75 | 0.97  0.72-1.31 |
| ≥75 | ≥75 | 0.69  0.36-1.31 |
